# Understanding how intermediaries connect adults to community-based physical activity: A qualitative study

**DOI:** 10.1371/journal.pone.0318687

**Published:** 2025-01-31

**Authors:** Megan O’Grady, Emer Barrett, Deirdre Connolly

**Affiliations:** 1 Discipline of Physiotherapy, School of Medicine, Trinity College Dublin, Dublin, Ireland; 2 Discipline of Occupational Therapy, School of Medicine, Trinity College Dublin, Dublin, Ireland; PLOS: Public Library of Science, UNITED KINGDOM OF GREAT BRITAIN AND NORTHERN IRELAND

## Abstract

Intermediaries facilitate connections to community-based services and supports, including physical activity and exercise groups, and are an emerging method to promote physical activity participation. However, their processes when establishing connections to community-based physical activities are unclear. The aim of this study was to explore the processes, practices, and procedures of Irish intermediaries when connecting people to community-based physical activity. This was a qualitative descriptive design study. Semi-structured interviews were carried out with n = 27 intermediaries from a variety of sectors—Health Promotion and Improvement [HPO], Local Sports Partnerships [LSO] and Social Prescribing [SP]. Four themes were identified using qualitative content analysis; 1) the processes of connecting to an intermediary, 2) connecting individuals to physical activity, 3) exiting from the intermediary service and 4) working in the local context. Intermediaries reported that they received referrals for individuals with physical, mental, and social health needs, but that referrals to improve physical activity specifically were low. They used a person-centred approach throughout their process, often addressing barriers to physical activity. However, only LSO and SP facilitated connections to physical activity, as HPO mainly focused on delivering smoking cessation support and services. Levels of support given and length of follow-up varied between LSO and SP, with the latter providing more intensive support. To facilitate their work, they developed extensive local knowledge and networks of partners, which enabled connections to a variety of community-based physical activities. Intermediaries may be an under-utilized resource to promote physical activity. Understanding the processes used in their interventions can inform future research, which is needed to investigate the effectiveness of intermediaries in improving physical activity levels and to inform future referral pathways.

## Introduction

Physical inactivity is defined as insufficient physical activity to meet current physical activity recommendations, i.e., at least 2 hours and 30 minutes per week of moderate intensity activity [1, 2]. Over a quarter of adults globally are failing to meet physical activity recommendations, and in Ireland this figure is as high as 54–61% [3–5]. To address the issue of physical inactivity, a comprehensive approach requiring multiple concurrent strategies is recommended, such as healthcare systems promoting physical activity, and community-based approaches like individually adapted health behaviour change interventions, social support interventions, and the creation of places for physical activity [6–10]. However, supporting physically inactive individuals to ‘bridge the gap’ between healthcare services and community-based physical activity services remains challenging.

Ireland’s publicly funded healthcare system is currently undergoing significant reorientation towards primary and community care settings, and towards chronic disease prevention and management in the community [11–13]. Examples of new services and programmes include primary healthcare community healthcare networks delivering services across populations of 50,000 people, chronic disease hubs providing specialist care services for individuals living with more complex chronic disease and multi-morbidity, and the Healthy Communities programme in areas across Ireland where health inequalities are most evident [12–14]. Engaging in physical activity is an essential component of chronic disease prevention and management, and community-based physical activity services align with the goal to ‘shift’ away from medical to community services.

Healthcare professionals have a responsibility to support their patients to be physically active, but health behaviour change can take time, ongoing follow-up, and appropriate resources. Healthcare professionals often report a lack of time to engage in meaningful and constructive dialogue around physical activity during appointments [15–17]. They can often work in less-than-favourable conditions, experiencing high work pressures, stress, and staff shortages [18–20], which may limit their capacity to offer ongoing follow-up and support [21]. In addition, healthcare professionals often perceive a lack of local physical activity services, opportunities or places to which individuals could be referred [20, 22, 23] or, they are unaware of existing community resources and services [24–26]. A 2013 survey of Irish healthcare professionals’ physical activity promotion practices found this was mainly performed on ad hoc basis, with a perceived lack of local services [21]. Almost ten years later in 2021, lack of time and awareness of local services remain key barriers to physical activity promotion in the Irish context [27].

An intermediary is considered an emerging method to address these gaps. An intermediary’s role is to act as a link person, or connector, to community-based physical activity, or other community resources, services and supports while offering increased time, follow-up, and personalised solutions to the needs of individuals referred to their services [28–33]. These are complex interventions, consisting of pathways with interacting elements and relationships between referring agents (often healthcare professionals), service users, intermediaries, and community-based physical activity, all of which take place within the local context and the context of the health needs of service users [28, 29, 31, 34]. Therefore, it is perhaps unsurprising that a scoping review carried out as part of this research identified significant gaps and heterogeneity in the descriptions of the processes undertaken by intermediaries when facilitating connections to community-based physical activity [35]. Many included studies did not report on ‘real-world’ intermediary services, and instead described a service that was designed and implemented specifically for a research trial. Therefore, information reported in these studies regarding referral, assessment and follow-up processes when connecting individuals to community-based physical activity, are unclear and may not reflect current practice.

Intervention identification and refinement are key phases of complex intervention development and evaluation [34]. Intermediaries could be a promising method to help address the problem of physical inactivity, particularly in Ireland where healthcare professionals find physical activity promotion challenging in practice. If the processes involved in the intermediary intervention were better understood across multiple contexts, future evaluation studies could then explore potential mechanisms of impact and outcomes [34, 36]. Therefore, the aim of this study was to explore the role of intermediaries in Ireland, in connecting adults to community-based physical activity, and to further define the processes, practices and procedures in the Irish context. This paper will discuss the findings in relation to each of the study objectives, which were:

i. To describe the pathways of connections from the referring agent to the intermediary;
ii. To explore the profile of people referred to an intermediary who are then connected to community-based physical activity services;
iii. To explore how intermediaries choose a suitable and appropriate community-based physical activity service for the person referred, and pathways of connections onwards from the intermediary to these services;
iv. To identify how intermediary services are established and embedded within the community.

## Methods

### Study design

This study used a qualitative descriptive design, to provide a rich, straight description of the experiences of study participants close to their own language [37]. Qualitative descriptive designs have been identified as a useful approach when research questions are focused on discovering the who, what, and where of events or experiences and gaining insights from informants regarding a poorly understood phenomenon [37–39]. Ethical approval for this study was granted from the Trinity College Dublin School of Medicine Research Ethics Committee (Application Number: 20220601) in July 2022.

### Participants

Based on discussions between the research team, literature review and consultations with the project advisory panel, three types of intermediaries were identified in Ireland—Health Promotion and Improvement Officers [HPO], Local Sports Partnership Officers [LSO] and Social Prescribing Link Workers [SP]. A brief description of each intermediary is provided, with detailed descriptions of these roles available in the supplementary material (S1 File). Participants were intermediaries aged >18 years old, employed in their role for ≥6 months and able to give informed consent. Exclusion criteria were retired workers or those on extended or maternity leave.

#### Health Promotion and Improvement Officers [HPO]

HPO act as advocates, both to build capacity within the Irish healthcare system and to ensure that available resources are used optimally to improve the health and wellbeing of the population. HPO work to target modifiable lifestyle risk factors such as smoking, alcohol consumption, obesity, and physical inactivity.

#### Local Sports Partnership Officers [LSO]

LSO support the development of opportunities to increase sport participation, and work to address barriers to physical activity participation. They also work to develop sustainable local leadership for sport within communities.

#### Social Prescribing Link Workers [SP]

SP design and co-produce personalised solutions so that people with social, emotional, or practical needs are empowered to find solutions which will improve their health and wellbeing, often using services provided by the community and voluntary sector.

### Participant selection

Recruitment for this study took place from the 7th of September 2022 until the 13th of December 2022. Gatekeepers and professional organizations (Health Service Executive Health and Wellbeing, Association for Health Promotion Ireland, Irish Social Prescribing Peer Network and Sport Ireland) were identified by the research team or by members of the project advisory panel, through either personal/professional networks, or publicly available information such as websites. Where contact details or a suitable person could not be found using publicly available information, the organizations were contacted for the details of a suitable person. Participants were invited to take part in the study via an invitation circulated through gatekeepers in the relevant professional organisations and via the research team’s social media accounts to maximise reach. Three rounds of invitations were sent over the recruitment period.

Purposeful sampling was employed to include ‘typical cases’ and ensure representation from all three types of intermediaries, wide geographical spread, and different levels of professional experience [40]. The research team planned to continue recruitment until ten of each intermediary type were recruited, or until no new analytical insights were emerging from interviews, whichever came first. This was determined by discussion between the research team. Intermediaries were recruited from a variety of sectors, and those recruited to this study were based in primary care, community healthcare networks, local health offices (where community health and personal social services are delivered), general practitioner offices, health promotion settings, community centres, local development companies (multi-sectoral partnerships that deliver community and rural development, social inclusion, and social enterprise services) local authorities and local councils (local authorities operate within specific geographic areas called local government areas, each of which has a council, and are responsible for the provision of public services). Participants gave written informed consent to participate.

### Data collection

Interviews took place from September 2022 to January 2023. Semi-structured interviews were conducted according to the participant’s personal preference–in-person, online (hosted via Zoom) or via telephone. All interviews were conducted using an interview schedule designed by the research team, which was informed by previous qualitative studies carried out with intermediaries [41–43], and the findings of the scoping review carried out as part of the research [35]. The schedule was also reviewed by the project advisory panel to establish credibility, with no changes suggested. Topics included the professional background of the intermediary, the profile of individuals referred to the intermediary, the processes of referral and assessment, the characteristics of onward connections to community-based physical activity, and how these services were monitored. This guide was designed to be iterative, with additional questions added as necessary in response to earlier interviews.

### Data analysis

Interviews were recorded and transcribed verbatim by a professional stenographer, or by using the auto-transcription feature on Zoom. Pseudonymized transcripts were analysed using an inductive qualitative content analysis approach [44], from January 2023 to August 2023. This methodology was chosen as it is a flexible but systematic coding and categorizing approach suitable for exploring large amounts of textual information [45–47]. In addition, it is well-suited for qualitative health-based research, analysing complex interventions, and for exploratory work in areas where not much is known about a topic [44, 45, 48]. This approach broadly involves three steps: 1) preparation, 2) organising, and 3) reporting [44]. Themes are developed by coding, development of preliminary themes or ‘categories’, relating categories/themes to published research and production of the written report [45].

#### Preparation

All interviews were conducted by the lead author, who is a female Ph.D. candidate, and this study formed part of her Ph.D. research. Field notes were taken during the interviews and formed the basis of ‘initial impressions’ which were used during update meetings between the research team. Additional preparation and familiarisation also occurred while listening to the audio recordings, transcribing interviews, and cleaning the transcripts. A personal research diary was kept by the lead author throughout the process of analysis. The purpose of this diary was to record any shifts in perspectives, establish credibility, record reflections on subjectivity and reflexivity, and to improve rigour.

#### Organising

Transcripts were read several times by the lead author to ensure familiarisation. Three rounds of coding were completed by the lead author. Analytical notes and open codes were made on the transcripts to describe all aspects of the content [49]. Over 650 codes were identified and coded using NVivo 12 and organised into clusters (groups of codes that share similarities [46]) using Microsoft Excel. Clusters were then grouped under generic categories, such as ‘promotion of the role’, ‘assessment’ and ‘local physical activity’. Categories in qualitative content analysis describe the manifest content of the data and are used at the beginning of the data analysis process to enter the abstraction process [46]. Categories relevant to the research objectives and the ‘steps’ of the process of the intermediary were selected and organised into themes by the research team.

#### Reporting

The findings of this study were generated by describing contents of the clusters within the identified themes [44]. Audit trails were kept ensuring dependability, and a sample audit trail for a cluster is included in the supplementary material (S2 File). An advisory panel, consisting of professionals from health promotion, local sports partnerships, and social prescribing services, were involved throughout the study. The panel advised on the study population, reviewed and gave feedback on study materials, and assisted with recruitment. A summarised version of the study findings was also reviewed by the panel to ensure credibility and confirmability. This study was reported using the COnsolidated criteria for REporting Qualitative research (COREQ) checklist [50].

## Findings

Interviews were carried out with n = 29 intermediaries (n = 11 SP, n = 10 HPO, n = 8 LSO). Interviews lasted 37 minutes on average (range 24–57 minutes). Fifteen interviews were conducted over Zoom, twelve over telephone, and two were conducted face-to-face at the intermediary’s place of work. Demographic information of participants is shown in Table 1. Two participants were excluded at the analysis stage, as it transpired during their interviews that they were working as volunteers in their current role. The decision was made to exclude these participants, as they would not be considered professionals and may lack the skills and resources required to deliver complex intermediary interventions [51, 52]. This resulted in 27 interviews included in the final analysis.

**Table 1:** Demographic information of participants (n = 27).

Type	Demographic	Result		
HPO (n = 9)	**Gender**	Female	6 (67%)	
		Male	3 (33%)	
	**Urban or rural**	Urban	4 (44%)	
		Rural	2 (22%)	
		Urban/rural	2 (22%)	
		Rural/urban	1 (11%)	
	**Setting of intermediary service** ^a^	Primary care	5	
		Health promotion office	2	
		HSE local health office	2	
		Community healthcare network	1	
		Freelance work in the community	1	
		Local authority	1	
	**Professional background** ^a^	Health and social care	6	
		Physical activity and exercise	4	
		Education	1	
	**Time in role (years)**	Range	1–18	
LSO (n = 8)	**Gender**	Female	6 (75%)	
		Male	2 (25%)	
	**Urban or rural**	Rural/urban	3 (37.5%)	
		Urban	2 (25%)	
		Urban/rural	2 (25%)	
		Rural	1 (13%)	
	**Setting of intermediary service** ^a^	County council offices	3	
		Community centre	2	
		Local authority	2	
		Sports partnership offices	1	
	**Professional background** ^a^	Physical activity and exercise	8	
		Education	1	
	**Time in role (years)**	Range	1–15	
SP (n = 10)	**Gender**	Female	10 (100%)	
	**Urban or rural**	Urban	6	60%
		Urban/rural	2	20%
		Rural	1	10%
		Rural/urban	1	10%
	**Setting of intermediary service** ^a^	Local development company	6	
		Family resource centre	2	
		Community centre	1	
		GP practice	1	
		Primary care	1	
		Resource centre	1	
	**Professional background** ^a^	Health and social care	8	
		Education	3	
		Private sector	1	
	**Time in role (years)**	Range	<1–15	

^**a**^Some intermediaries were based in more than one setting, or had backgrounds in multiple sectors. Urban/rural indicates the intermediary worked in a mostly urban setting. Rural/urban indicates the intermediary worked in a mostly rural setting. Physical activity and exercise professional background includes any work in the physical activity sector, including sport and private fitness sectors. Health and social care professional background includes healthcare, social care, youth work, mental health, community development, health promotion and disability/inclusion roles. Education professional background includes any teaching, coaching or guidance role. Abbreviations: GP–general practitioner, HPO–health promotion and improvement officer, HSE–Health Service Executive, LSO–local sports partnership community development officer, SP–social prescribing link worker.

**Fig 1:**
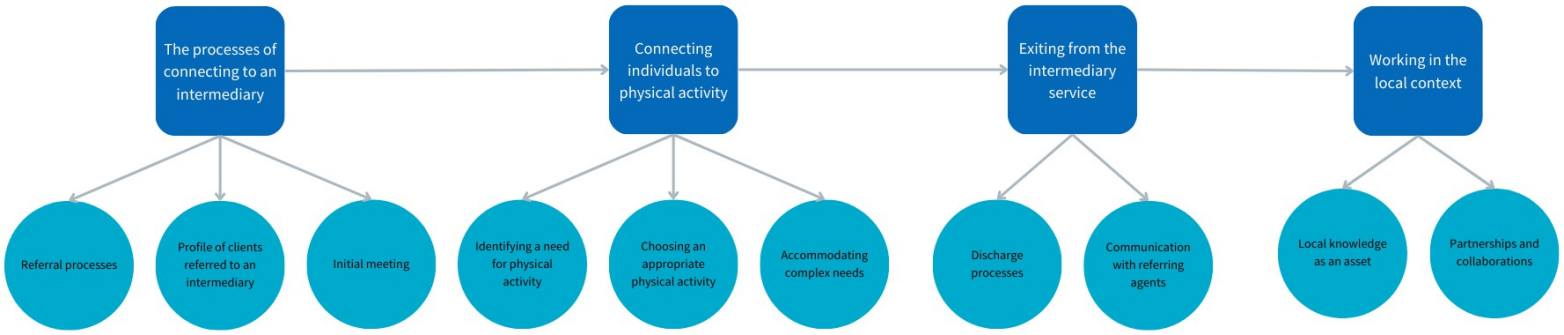
Themes and sub-themes.

Four themes were identified: the processes of connecting to an intermediary, connecting individuals to physical activity, exiting from the intermediary service, and working in the local context. Together, these themes discuss the processes of referral, assessment, and follow-up when intermediaries connected individuals to community-based physical activity. They describe how intermediaries selected appropriate community-based physical activities based on the referred individual’s needs, as well as how individuals left the intermediary service after being connecting to an activity. Finally, the broader contextual factors that influenced the work of intermediaries are presented. A detailed description of each theme and sub-theme is given under each theme heading, and themes and sub-themes are shown in Fig 1.

### The processes of connecting to an intermediary

This theme describes how and why individuals were referred to an intermediary, as well as the demographic and health characteristics of these individuals. This theme also describes the initial meeting between intermediaries and service users. Three sub-themes were identified: ‘referral processes’, ‘profile of individuals referred to an intermediary’ and ‘initial meeting’.

#### Sub-theme: Referral processes

Referring agents varied widely both within and between the three intermediary types. All types of intermediaries worked closely with healthcare services who were identified as a major source of referrals to HPO, SP and to a lesser extent LSO. In addition, referrals would often come directly from the community itself, through local community, voluntary and charitable sectors or through friends and family members of the person referred.

*“Individuals can self-refer, it could be a GP, it could be community mental health, it could be private counsellors, physios, OTs [occupational therapists], public health nurses… I would get friends referred to me or you know a Granny might refer her grandson you know just word of mouth as well*.*”*

*P23, SP*

Reasons for referral could be categorized broadly as after a change in life circumstances or to address a pre-existing health issue.

*“It would be people with loneliness, isolation, physical problems, mental health problems, emotional needs, practical needs… there’s no one type of person that gets referred into us all the time because it just changes*.*”*

*P27, SP*

*“Those who are excluded or marginalized… either by socioeconomic status, either by location of where they live*.*”*

*P10, HPO*

Self-referrals were also reported by all intermediary types, and this was the most common route of referral to LSO. A minority of intermediaries across the three types reported that individuals were referred by a referring agent specifically to improve fitness, physical activity levels or to connect to a community-based physical activity service.

*“I’ve never actually had a referral where someone has outlined a person’s physical inactivity*.*”*

*P27, SP*

#### Sub-theme: Profile of individuals referred to an intermediary

Referred individuals (service users) tended to be mid- to older-aged adults. According to intermediaries, less men accessed their services or engaged in physical activity groups.

*“Now I’d say off hand the majority of those would be female, I’d say if you had 50 males [out of >500 participants] out of that you’d be doing well*.*”*

*P08, LSO*

Intermediaries reported that service users commonly presented with co-morbidities, such as cardiovascular, respiratory, neurological, and musculoskeletal disease.

*“I’d be getting general referrals from adults… they’d have a wide range of health conditions… diabetes, obesity, osteoporosis, osteopenia, people with high blood pressure, high cholesterol*.*”*

*P11, LSO*

Mental health issues were also frequently reported, alongside social isolation. These issues may have been reported at the time of referral but were often observed by intermediaries during the assessment or follow-up periods.

*“Typically people who I see are vulnerable*. *They are isolated in the community*. *They mightn’t be leaving their house very often*. *They don’t have many connections in the community*.*”*

*P18, SP*

Intermediaries also reported that service users frequently had difficulty with mobility, impaired balance, and falls.

*“People who are mobility challenged… You’ve older people who reach the stage where they can no longer drive, or they’re physically not strong enough to take public transport*.*”*

*P16, SP*

#### Sub-theme: Initial meeting

For LSOs, the initial contact with service users took place over phone, email, or at the first session of a community-based physical activity. This was an opportunity to give them information about other physical activity services that were currently available, and to develop a rapport.

*“They get to know you, you know, so you strike up a relationship with them and stuff like that*. *So you know the trust is there*.*”*

*P22, LSO*

No explicit assessments were carried out by LSO. A longer, more in-depth assessment was carried out by HPO and SP. Intermediaries started by introducing or explaining the service and their role. The discussion would then shift to the service user, to discuss lifestyle behaviours including physical activity, smoking, diet, sleep, and other physical health issues.

*“I’ll talk about any issue and guide people to any service [physical activity, weight management, bereavement support] and I’d have no problem about that*.*”*

*P25, HPO*

General wellbeing, including mental health, social and community connectedness, and other psychosocial needs were also discussed, but only by SP. The ethos of this first meeting was person-centred and holistic, even if processes varied from one SP to the next.

*“Even the fact they’ve come, and you’ve listened to them*. *One of the best things we can do is sit, and we can give them time… a minimum of 45 minutes, mostly maybe the hour, or an hour and 15 minutes*. *And so that it’s very powerful*.*”*

*P28, SP*

Some services aimed to complete standardised outcome measures during the initial meeting and again at the time of exit from the service.

*“At that initial meeting we would run some basic outcome measures to establish a baseline*. *We’d use the Warwick [Edinburgh Mental Wellbeing Scale], and we’d use the WHO-5… just to get a sense of the person, what their hobbies have been in the past, what’s brought them to this point, and to establish that rapport*.*”*

*P09, SP*

### Connecting individuals to physical activity

This theme describes the processes undertaken by intermediaries following the initial meeting with a service user. It describes how intermediaries identified physical activity as a need, and how a community-based physical activity was selected based on the service user’s needs. This theme also describes the practices of intermediaries during the ‘follow-up’ period after the initial meeting, when establishing and maintaining connections to community-based physical activity services. Three sub-themes were identified: ‘identifying a need for physical activity’, ‘choosing an appropriate physical activity’ and ‘accommodating complex needs’.

#### Sub-theme: Identifying a need for physical activity

While physical inactivity was often not the primary reason why they were referred, during the assessment service users could reveal physical inactivity as an issue that they wished to address. The assessment could then also be used to identify barriers to physical activity and set goals in relation to engaging in physical activity.

*“If they highlight it as a goal… But for a lot of people it would be that they highlight physical activity… “Oh, I want to lose some weight”, or “I want to do some exercise to feel better”, or “my doctor told me because of my condition I need to do exercise and I’ve never gone to a gym, and I don’t know what to do”*.

*P09, SP*

All intermediaries identified that physical and mental co-morbidities often negatively impacted on service users’ ability, readiness, or interest in engaging in physical activity. Intermediaries reported other barriers to engaging in both physical activity and the intermediary service generally, such as mobility and physical limitations, social isolation, family and employment commitments, socio-economic circumstances, and gender.

*“Say you had somebody with diabetes or some chronic health condition, they are so focused on their day to day and managing it from a very I suppose basic level that they don’t maybe see themselves as somebody that would benefit from movement*.*”*

*P03, SP*

Despite these barriers, all intermediaries recognised the potential benefits of physical activity for health, and that some service users needed additional supports to become more physically active.

*“Other ones that I know will have something to do with exercise could be… Any of the chronic health stuff*. *Then I’d kind of be like, okay*. *Exercise*. *Like in the back of my head*.*”*

*P18, SP*

As such, intermediaries would provide behavioural support, such as reassurance and motivational support.

*“So the odd time I would still get a phone call or an email, “am I suitable for this programme?” you know, “would I be able for it?”… More often than not once they know that they are physically well then you’d encourage them to sign up and to get involved… The reassurance piece I suppose is a big thing in our space here, I think*.*”*

*P24, LSO*

This could be in combination with practical support, like attending the first session of the group (LSO and SP) or being available as needed throughout the process of connection (SP only).

*“Other people they’re like, ’no, can you just meet me for a coffee before I go the next time, because it was really scary’… so they might need some continued support*.*”*

*P18, SP*

In terms of length of follow-up, for LSO contact generally ceased once service users had attended a community-based physical activity.

*“You know generally 99% of people that contact us will generally turn up to our programmes and they generally will partake*.*”*

*P22, LSO*

HPO did not actively connect service users onwards to physical activity. They explained that they mainly received referrals related to their primary role as smoking cessation officers. These officers were often located in primary care services related to chronic disease management.

*“In terms of me meeting the public the closest access I would have is in our stop smoking clinic… that’s the closest and most tangible time I’ll meet people*.*”*

*P4, HPO*

For SP it depended on the service user and their needs and ranged from one session to multiple sessions over a longer period of time.

*“Some people who want to work on their physical health are just looking for information on what’s available to them and that’s a simple case of signposting and maybe a bit of motivational support in our chat*.*”*

*P19, SP*

#### Sub-theme: Choosing an appropriate physical activity

All intermediaries discussed how service users’ preferences and interests were used to guide selection of a suitable community-based physical activity service.

*“I’m not sure if I agree with that question about me deciding on a group, because again it very much depends on the person themselves*.*”*

*P19, SP*

Service users were connected to physical activities that would be enjoyable and suited to their needs, such as their physical health, physical fitness, or psychosocial needs.

*“It would be kind of thinking about what’s on, thinking about the person’s ability, and what they’re going to get the most enjoyment at, because it’ll suit them*.*”*

*P18, SP*

Only one LSO intermediary explicitly said that they would choose a community-based physical activity service based on the quality of the service. The physical activity instructor was considered by intermediaries as an important feature in the process of encouraging service users to take part in a group, especially as the intermediary began to step back and the frequency of contact began to decrease.

*“The social prescriber cannot become a befriending service to the point where we are accompanying somebody consistently, it would be too much*. *So I have to trust… the facilitator of that programme, is able to take up that hand-holding to a degree*.*”*

*P27, SP*

LSO and SP believed that physical activity instructors should be knowledgeable about physical activity and exercise and be skilled in adapting and delivering exercise suitable to the service user’s needs.

*“There’s a health screen where we find out any information in advance*. *So the instructor’s prepared and know[s] about the person coming in and can prepare… the class that they need*.*”*

*P08, LSO*

#### Sub-theme: Accommodating complex needs

To accommodate physical health needs, all intermediaries highlighted the importance of seeking medical clearance before the service user engaged in physical activity. SP reported they often sought medical clearance for service users with more complex health needs or suggested medically supervised or condition-specific physical activity groups.

*“I just had a lady in recently who had fibromyalgia and she… had about 12 different things and she was referred by a physio*. *And what I would… tell her what’s available but I will be saying to her you need to check back in with the physio before you would participate with any of these activities*.*”*

*P23, SP*

To support older adults to engage in physical activity, intermediaries recommended groups specifically designed for older adults or focused on falls prevention and balance.

*“So we do chair-based exercise classes, but it’s very functional… it’s focused on falls prevention, a lot as well*.*”*

*P29, LSO*

All intermediaries recognised the potential digital literacy needs of older adults, and offered support to those that were less computer-literate.

*“But still a lot of older people are struggling with the online [registration] so we still facilitate that in terms of the walk-ins or posting out the registration forms as well*.*”*

*P08, LSO*

Community-based physical activity services offered socially isolated service users the opportunity to connect and build wider peer support and community networks.

*“I kind of approach it with a person like… a balanced body, a balanced mind… I try and suggest something physical… Usually the physical thing is part of a group… it gives them that regularity of contact with a bunch of people*.*”*

*P07, SP*

For service users of SP that were not ready or interested in engaging in physical activity, intermediaries recognised this had the potential to change over time.

*“But I suppose it’s having an awareness that sometimes people aren’t at the place to engage, and sometimes it is planting that seed*.*”*

*P09, SP*

To assist service users in making this change, SP intermediaries reported several strategies. They may have suggested an alternative community service to address barriers to being physically active (for example, a service user with weight management difficulties attending a healthy eating programme), incorporated physical activity into other activities (for example, through volunteering) or adjusted their level of support accordingly.

*“I think a lot of the time when people are reluctant, when there is something that they want to engage with but there’s this bit of a barrier there, that offer of that support can be really, really helpful for people to actually take that first step and become more engaged*.*”*

*P19, SP*

### Exiting from the intermediary service

This theme describes how service users were ‘discharged’ or exited from intermediary services, as well as processes used by intermediaries to communicate back to referring agents. Two sub-themes were identified: ‘discharge processes’ and ‘communication with referring agents’.

#### Sub-theme: Discharge processes

Both LSO and SP spoke about service users continuing to engage in community-based physical activity services after the connection was made, which was taken by many intermediaries as a proxy measure of improved physical activity. However, this was rarely formally measured.

*“So we’re always trying to find a way for them to access physical activity… whether they stay with us or not… We don’t evaluate everything, because one, we wouldn’t have time otherwise, we’d spend most of our time evaluating*.*”*

*P01, LSO*

The ’discharge’ process varied from service to service and across intermediary types, but the person-led ethos to assessment and follow-up carried through to the exit process. Generally, the exit process began once service users had been connected successfully to a group or were deemed to be more socially connected.

*“Typically, when that happens you’ll see other things in their life start going*. *So when you ring them for the follow up after they’ve been engaged in something, they’ll be like, yeah, I’ve been going to that course*. *Yeah, I also went out to meet my sister for lunch, and I haven’t seen her in 6 months*.*”*

*P18, SP*

Several SP had no formal ‘discharge’ or exit process, with some intermediaries reporting that they had service users who had never stopped engaging with the service.

*“I’m six and a half years now in this post and there are some people that I am seeing, maybe every couple of months from the beginning*.*”*

*P21, SP*

LSO similarly spoke about service users returning to their service and participating in the same groups. This idea of ‘repeat attenders’ was identified by both SP and LSO.

*“If they just feel comfortable at that level, and you know, they don’t want to go on to anything else… they want to stay in that safety net of the Men on the Move group, and it’s a social element for them*… *I think that’s totally up to them*.*”*

*P02, LSO*

#### Sub-theme: Communication with referring agents

The final step in the process was feeding back to the original referring agent, if any. Only some referring agents requested feedback on service users’ progress, but in other services providing feedback was not common practice.

*“What I usually generally do is I don’t divulge details but I just inform the referral agent of the person’s engagement*. *So, a general email then to follow up and say that your referral engaged in the programme and they are engaging well or they’re not*.*”*

*P03, SP*

### Working in the local context

This theme describes the availability and types of community-based physical activity services within the intermediaries’ local community, and the contextual factors that facilitated their knowledge of these services. Two sub-themes were identified: ‘local knowledge as an asset’ and ‘partnerships and collaborations’.

#### Sub-theme: Local knowledge as an asset

Having a knowledge of currently available, local, community-based resources, services, and groups (local knowledge) was of significant benefit and a key component of the intermediary process. Intermediaries spoke about both service users and referring agents valuing the intermediary’s local knowledge.

*“Especially because it’s local, they know that you are working in that area, they know that it’s local to them, it’s not somebody ringing from [areas] or outside… they’re ringing because they’re looking for a local programme… they’re happy about that you know*.*”*

*P22, LSO*

However, building this local knowledge was often labour intensive. It was conducted and driven by the intermediaries themselves, and many spoke of the difficulties and challenges of keeping this up to date.

*“I would say the most up to date resource is in my own head [laughing] because… if I was to have an up-to-date resource at my fingertips it would just be a 24–7 job*.*”*

*P19, SP*

There was a large variety of community-based physical activity services available in the intermediaries’ local communities.

*“But I have to say, when I look at [city] compared to other cities, I do think we are very well resourced in terms of options for physical exercise, especially for people who would be intimidated by the idea of exercise, and would never consider themselves athletes, there are so many non-intimidating beginner options, and welcoming options that people can access*.*”*

*P09, SP*

Some of the more popular choices were dancing, chair-based exercise, local gyms and leisure centres, yoga and tai chi in addition to sports, gardening and water-based activities. Walking groups were by far the most reported activity by intermediaries and were a popular activity amongst service users.

*“People love walking*. *A lot of people want to do walking, walking groups… There’s a very well-established walking group in one of the [GP] practices, and lots of people from that practice want to join the walking group, so I get referrals to see them, and then part of that referral would be to introduce them to the walking group*.*”*

*P18, SP*

#### Sub-theme: Partnerships and collaborations

In addition to developing local knowledge, intermediaries spoke of an ongoing process of utilising and building networks of partnerships and collaborations within their community and local context. These partnerships often developed naturally over time and constituted a ‘coming together’ of partners working in the same area.

*“There’s no specific area where you say, oh, yeah, we have partnership, and this is where it started*. *It’s built up over time… they know this local sports partnership is there, and we know that they’re there in a sense*.*”*

*P29, LSO*

Intermediaries described partners becoming aware of the others’ presence and specific attributes of the service, and learning how each partner could help to implement health and physical activity policy and strategy within the local context.

*“So you are now linking in with the HSE [Health Service Executive], Sláintecare, [local council], other departments so we’re all kind of starting to work together or meet or share details or network or things like that*. *It’s kind of growing… it kinda wasn’t there in the past*. *You may have felt you worked on your own*.*”*

*P22, LSO*

Intermediaries explained how partners brought specific skills and knowledge to the partnership, enabled cross-referrals where appropriate, and provided important resources such as equipment, funding and venues for community-based physical activity services.

*“It’s really about linking in with the likes of the development companies… family resource centres… local GAA [Gaelic Athletic Association] clubs, your community groups, your over 55s groups, because these people, they have people on the ground they linked in with… we might find it hard to infiltrate these rural settings, a little hamlet, or whatever, so it’s important to build on what’s already there*.*”*

*P20, HPO*

Intermediaries working in rural areas reported significant, unique challenges, with scattered hubs of community resources and services and service users often presenting with rural isolation. Issues that likely were contributing factors to rural isolation also made it difficult to access physical activity services, such as lack of transport.

*“Travel is a big issue in the rural areas and we would see that in [county]… so it’s great if you can go into the community whereas if you’re doing a [exercise group in a] town over approach you’re cutting off a lot of people that really need to be active*.*”*

*P08, LSO*

## Discussion

This study aimed to gain insight into the processes, practices, and procedures of intermediaries in Ireland, in connecting people to community-based physical activity. This study described the steps of these processes of three types of intermediaries: Health Promotion and Improvement Officers [HPO], Local Sports Partnership Officers [LSO] and Social Prescribing Link Workers [SP]. While a number of commonalities were identified across the three types of intermediaries, processes were varied and heterogeneous.

Processes and patterns of referrals were heterogeneous between types of intermediaries, as may be expected based on the differences in job remit. Our scoping review found the most common sources of referral were from primary care staff, general practitioners or as part of a research study [35]. However, intermediaries in this study reported that referrals were accepted from almost every source in the community, with few restrictions on who could refer and how. This highlights the accessible and open nature of these services, especially the acceptance of self-referrals, and means that their services are available to individuals who may not present to healthcare services or settings. The fact that many intermediaries were based in non-healthcare settings may have also contributed to the accessibility of their services. Intermediaries who participated in this study were based in community centres, local development companies, local governments and councils. Intermediaries therefore have the potential to reach many population groups living within the community to help improve their physical activity levels, including those who may experience increased barriers and inequalities in accessing healthcare services.

A major finding was that while HPO received referrals for community-dwelling adults, they did not typically refer these service users onwards to community-based physical activity. However, the remit of a HPO in Ireland is very broad. They are expected to deliver health and wellbeing training programmes to health service staff, provide knowledge and expertise, and support different models of care within the health service to deliver health and wellbeing gains for the population [53]. This is in addition to their work providing one-to-one smoking cessation services. The current focus of the role on smoking cessation may reflect a key implementation or policy area at the time of this study [54]. While physical activity promotion may simply not be a priority at present, priorities could shift and change over the coming years.

Both SP and LSO reported receiving low numbers of referrals specifically to increase or improve physical activity. SP in this study tended to receive referrals for social reasons, similar to many social prescribing services across the world [55, 56]. Conversely, while individuals were referred or attended LSO exclusively to participate in their physical activity groups, this was mainly through self-referral. Despite low numbers of formal physical activity referrals to both SP and LSO, many intermediaries spoke about assessing the service user’s physical activity levels, their interest in physical activity, and then connecting service users to and having them engage in community-based physical activity. This appears to represent a disconnect between the reason for referral and what the service user reports they want to address [57]. It also highlights intermediaries’ awareness of the potential benefits of physical activity in addressing and improving multiple aspects of health. Overall, this study suggests that formal referrals to both LSO and SP to engage in physical activity could potentially be improved.

This study found that individuals accessing intermediary services appear to be experiencing poorer physical, mental, and social health and that intermediaries tried to accommodate these needs when connecting service users to suitable community-based physical activity. This is an important finding given the context of this study. Data collection for this study was carried out in 2022, when many intermediary and community-based physical activity services were resuming or reopening after periods of Covid-19 associated lockdowns. Lockdowns had detrimental effects on the ability and opportunity for individuals to engage socially, with many experiencing social isolation [58–60]. Greater social isolation in older men and women is related to reduced objective and subjective physical activity and greater sedentary behaviour [60–62].

A person-led approach was evident across all intermediary types in terms of assessment, follow-up and in choosing a community-based physical activity service for the service user, regardless of the length of their intervention. This was true even for LSO who reported more limited time with the service user, in contrast to SP who may remain in contact with a service user over a period of months or even years. In the Let’s Get Moving physical activity pathway, designed for the UK national health service, follow-up is recommended at three and six months after a physical activity intervention [63]. Longer periods of follow-up or more intensive follow-up have been identified as potentially successful components of physical activity interventions [64, 65].

However, it is unclear whether different referral patterns, assessment and follow-up practices influenced the intervention. For example, the optimum number and duration of contacts to initiate and maintain participation in physical activity remains unclear [15], particularly for service users with low agency or more complex health needs or issues. These service users may require more support and more intense interventions [51, 66–68]. This study identified that SP not only work with service users with complex needs but provide ongoing support to service users who were not ready to change their PA behaviour. Unfortunately, the interview schedule was not designed to explore how intermediaries may have handled resistance or built motivation over time. Future studies could take into consideration the findings of this study regarding the length of follow-up provided by each type of intermediary, as this may be an area of further research i.e., does more support throughout this process, over a longer time, equal more successful connections to community-based physical activity, and who would benefit from this more intensive support?

This study identified that intermediaries initiated and maintained participation in physical activity for service users with poorer health. This finding is of note, given the recent move to enhance community-based chronic disease prevention services within the Irish healthcare system. The need for enhanced supports to improve self-management skills for people living with chronic disease had also been recognised at a national level, with roll-out in 2021 of integrated care teams, chronic disease hubs and chronic disease models of care [13, 69]. Self-management involves the individual being an active participant and decision-maker regarding their health e.g., eating a healthy diet, medication compliance and participating in physical activity [70, 71]. The Irish healthcare system was and is undergoing major reform as part of the Sláintecare initiative, involving significant development and expansion of primary and community care services [11]. This echoes the call for community-based approaches to improving physical activity, where physical activity initiatives and programmes are offered where people live, work and engage in recreation [6]. This supports the need for community-based services, such as those provided by intermediaries, to support those living with social isolation, poor physical health, and the negative sequalae of these.

By facilitating and encouraging service users to participate in physical activity, there is potential for two distinct roles for intermediaries: one of primary prevention of ill-health, and one for secondary prevention of complications resulting from ill-health. However, while some intermediaries who took part in this study had previous experience or further training to support them working with people with complex needs (Table 1) this was not the case for every participant. The interview schedule was not designed to explore intermediaries’ perceptions of working with service users with more or less complex needs, so it is unclear whether all intermediaries felt comfortable and competent delivering interventions for these service users. Healthcare professionals often report a need for appropriate education and continuous training when promoting physical activity [17, 23, 27]. Intermediaries could perhaps therefore benefit from additional training to provide the optimal support to facilitate behaviour change for these service users, and to ensure selection of suitable physical activity services for these service users.

This study also explored how intermediaries chose a suitable and appropriate community-based physical activity service for service users. While this process was practical (e.g. medical pre-clearance, difficulties using online registration systems) and person-led (based on the individual’s abilities and interests), local knowledge was a key factor in this decision. However, the time-consuming nature of maintaining this knowledge was highlighted by many intermediaries in this study. Bar one participant, no intermediary interviewed for this study mentioned quality of the physical activity service as a factor in their decision to recommend a service. This may be in part due to an already demanding schedule keeping up to date with local community resources and working with pre-existing service users (for long periods of time, as evidenced by the lack of formal exit processes), but also the fact that outcomes in relation to onward connections to community-based physical activity were poorly captured.

Evaluating outcomes using appropriate measures would allow intermediaries to appraise the success of their onward referrals, and/or quality of the physical activity service. It may be unreasonable to expect evaluation at that level without further support and resources and highlights a potential area for further evaluation and research collaboration. For example, community-based physical activity providers could evaluate and feedback to intermediaries. Having more robust pathways and procedures in place for the evaluation of physical activity outcomes and of the physical activity groups being accessed by service users, may help to improve service user outcomes as well as help to reassure potential referring agents as to the safety and quality of the physical activity being delivered, helping to improve formal referrals.

Several important contextual factors were identified which influenced the processes of intermediaries. A commonality across all categories of intermediaries was building knowledge of local physical activity services, which was found to be a key input in the intermediary intervention. Other studies have also noted the importance of this local knowledge, and the importance of the intermediary’s own personal skills in developing and maintaining this knowledge [56, 72–74]. Intermediaries in this study were based in a variety of central community locations and this, alongside dedicated time and resources to build their local knowledge, means they are likely best placed to recommend individualised physical activity and wider health and wellbeing resources specific to their local context. However, one limitation of the reliance on intermediaries to gather and update this information individually is inconsistent or outdated resources, or services being missed due to limitations of their awareness [75].

Another commonality for all intermediary types was their commitment to building and maintaining partnerships and networks of collaborators. Intermediaries reported that partnerships allowed them to connect service users to appropriate and properly resourced physical activity services. Development of effective partnerships and referral pathways relies on careful selection of partners who will complement each other, who will strengthen the partnership’s goals and provide effective and efficient services to meet the needs of service users [76–78]. This study found intermediaries used a ‘bottom up’ approach to building partnerships and collaborations, with active ‘seeking out’ of local community partners. This is particularly relevant in the context of the Sláintecare roll-out, as a key focus is partnering with local agencies in order to target specific health and wellbeing needs in the local community [11, 12].

## Limitations

This study has a number of limitations. The sample was self-selected, and intermediaries who participated in this study tended to have professional backgrounds in health and social care and/or physical activity. Our sample could therefore represent those with a professional interest in physical activity promotion and not be representative of the processes of intermediaries generally. We aimed to recruit participants from the entire Republic of Ireland, but certain areas of the country were underrepresented, for example, counties in the midlands and south of the country where intermediaries may report different processes for connecting individuals to physical activity services. SP and HPO participants tended to have limited experience in their role, with the modal length of time in the role being one year. However, this is likely a reflection of new investment by the national health service in intermediary services, with many of these roles being created within the last few years. As many of these new roles are still in development, their full scope and remit will continue to evolve over time. Therefore, this study represents an overview of the processes and procedures relevant to the intermediaries who participated in this study which is likely to grow and progress over time in response to local needs, funding, and policy directives. A further limitation was not including referring agents and/or service users which limits insights into their experiences of engaging with this intervention.

## Conclusions

Local Sports Partnership Officers [LSO] and Social Prescribing Link Workers [SP] are two types of intermediaries in Ireland that facilitate connections to community-based physical activity, by providing different levels of support, whereas Health Promotion and Improvement Officers [HPO] did not actively connect service users onwards to physical activity. Despite this, referrals from healthcare and other professionals to LSO and SP to specifically improve physical activity are low. Considering the high levels of physical inactivity in Ireland, and the challenges associated with connecting physically inactive people to community-based physical activity, intermediaries may be an under-utilized resource to increase physical activity. This study identified and described the steps involved in the processes of establishing and embedding intermediary services within the context of their local community, and pathways of connecting individuals to intermediaries and onwards to community-based physical activity. This is an important step to improve evaluation efforts, synthesis of evidence and the planning of future trials. Further studies are needed to investigate the impact of interventions provided by intermediaries, across the continuum of support provided, and using appropriate outcome measures, to capture physical activity levels of service users. Future trials can now focus on other key phases of complex intervention evaluation, such as feasibility and evaluation [34], and a pilot feasibility trial is currently underway [79].

## Supporting information

S1 File Detailed descriptions of intermediary roles. A detailed description of the health promotion and improvement officer, local sports partnership officers and social prescribing link worker roles in Ireland.
(DOCX)

S2 File Sample audit trail. A sample audit trail for the cluster ‘mental health issues negatively impact engagement’, part of the theme ‘connecting individuals to physical activity’.
(DOCX)
