# Supplementary material for: Understanding how intermediaries connect adults to community-based physical activity: A qualitative study
Source: PLoS One. 2025 Jan 31;20(1):e0318687. doi: 10.1371/journal.pone.0318687 (PMC11785267; doi:10.1371/journal.pone.0318687)
Supplement: S1 File — A detailed description of the health promotion and improvement officer, local sports partnership officers and social prescribing link worker roles in Ireland. (DOCX) [file pone.0318687.s001.docx]

**S1 File. Detailed descriptions of intermediary roles**

**Health promotion and improvement officers**

Health Promotion and Improvement Officers act as enablers, negotiators, and advocates to build capacity within the health service and with external stakeholders to improve the health and wellbeing of the population. They aim to embed preventative health measures and messages into health service delivery, targeting modifiable risk factors such as smoking, alcohol consumption, physical inactivity and obesity [1, 2]. Some officers also operate as ‘smoking cessation officers’, with a remit to assist smokers through the process of a quit attempt, providing behavioural support, pharmacological information/prescription and/or referral for prescription to effectively treat tobacco addiction. Significant investment has been made into the development and embedding of the Health Promotion service as part of the Healthy Ireland Strategic Action Plan 2021-2025, developed in order to facilitate the implementation of the Healthy Ireland framework [3]. As a result, many new posts have been created to support health promotion, and these were being filled at time of this study. It was estimated that N=246 health promotion officers were working across the country at the time of the study (personal communication via email with community health organisation Health and Wellbeing managers, October-November 2022).

**Local sports partnership officers**

Sport Ireland is the authority tasked with the development of sport in Ireland, and operates through its network of 29 local sports partnerships across Ireland [4]. Local Sports Partnership community sports development Officers support the development of opportunities to increase sport and physical activity participation, and develop sustainable local leadership for sport within communities. They foster and encourage a culture of active participation, addressing barriers to physical activity participation for those who experience disadvantage for any reason [5, 6]. As such, they are a key implementation partner of the National Sports Policy 2018-2027 [7]. The first eight local sports partnerships were launched in May 2001, with four more partnerships launched in June 2002 [8]. The community sports development officer programme was first established in 2016, to strengthen local delivery and capacity of the local sports partnership. A nationwide roll out of the programme was carried out in 2019, after which one officer was in situ in all 29 local sport partnerships across the country [6]. At the time of this study, all 29 posts were filled (personal communication via email with Sport Ireland, June 2022).

**Social prescribing link workers**

Social Prescribing Link Workers design and co-produce personalised solutions so that people are empowered to find solutions which will improve their health and wellbeing, self-management skills and social connectedness. The ultimate aim is to achieve this by connecting people to community groups, organisations and statutory services for practical and emotional support [9, 10]. The government of Ireland has committed to the expansion and roll-out of social prescribing, and this is an action in many recent health strategies and polices, including the Department of Health mental health strategy, the Sláintecare Implementation Strategy and Action Plan 2021-2023, the Healthy Ireland Action Plan 2021-2025, and the Health Service Executive Social Prescribing Framework [3, 11-13]. Some social prescribing services had existed for years prior to this more recent enhanced investment and roll-out, for example in counties Mayo and Donegal [13]. At the time of this study, N=61 link workers were working across the country (personal communication with Irish Social Prescribing Peer Network via Zoom, May 2022).

**References**

1. Health Service Executive (HSE), Health Business Services (HBS). Health Promotion and Improvement Officer (HP&IO) Smoking Cessation (Grade VI) Job Specification Terms and Conditions. 2020 [cited 2024 April 2]. Available from: <https://www.hse.ie/eng/staff/jobs/job-search/management-admin-ict/20hpsc-job-specification.doc>.

2. Health Service Executive (HSE). Grade VI Health Promotion and Improvement Officer (HP&IO) Health Promotion and Improvement Job Specification & Terms and Conditions. 2022 [cited 2024 March 29]. Available from: <https://www.rezoomo.com/contentFiles/jobs/35120/attachments/13706_DNCC2022147%20Health%20Promotion%20and%20Improvement%20Officer%20Job%20Specification.pdf>.

3. Government of Ireland. Healthy Ireland Strategic Action Plan 2021–2025 Building on the first seven years of implementation. 2021 [cited 2024 April 2]. Available from: <https://www.gov.ie/en/publication/441c8-healthy-ireland-strategic-action-plan-2021-2025/>.

4. Sport Ireland. Local Sports Partnerships 2023 [cited 2023 August 9]. Available from: <https://www.sportireland.ie/participation/local-sports-partnerships>.

5. Limerick City and Council, Sport Ireland Local Sports Partnership, Limerick Sports Partnership. Limerick Local Sports Partnership Community Sports and Physical Activity Development Officer CSDO Job Description. 2021 [cited 2024 April 2]. Available from: <https://limericksports.ie/wp-content/uploads/2021/03/Community-Sports-Development-Officer-Position-2021.pdf>.

6. Sport Ireland. Community Sports Development Officers Evaluation Report 2020. 2021 [cited 2024 April 2]. Available from: <https://www.sportireland.ie/sites/default/files/media/document/2021-01/csdo-evaluation-2020-full-report.pdf>.

7. Government of Ireland. National Sports Policy 2018 - 2027. 2019 [cited 2024 March 29]. Available from: <https://www.gov.ie/en/publication/aaa7d9-national-sports-policy-2018-2027/>.

8. Westmeath Sports Partnership. Background 2023 [cited 2023 July 4]. Available from: <https://www.westmeathsports.ie/about/background/#:~:text=Background%20to%20the%20Local%20Sports%20Partnership%20(LSP)%20Initiative&text=The%20first%20eight%20partnerships%2C%20in,Dublin>.

9. Southside Partnership Dun Laoghaire. Social Prescribing Link Worker Job Description. 2020 [cited 2024 April 2]. Available from: <https://ildn.ie/wp-content/uploads/2020/01/Social_Prescribing_Link_Worker_January_2020.pdf>.

10. Wexford Local Development. Job Description Social Prescribing Link Worker. 2021 [cited 2022 November 1]. Available from: <https://www.wld.ie/careers-2/job-description-social-prescribing-link-worker/>.

11. Department of Health. Sharing the Vision A Mental Health Policy for Everyone. Dublin, Ireland: 2020 [cited 2024 March 29]. Available from: <https://www.gov.ie/en/publication/2e46f-sharing-the-vision-a-mental-health-policy-for-everyone/>.

12. Department of Health. Sláintecare Implementation Strategy & Action Plan 2021 — 2023. 2021 [cited 2024 March 29]. Available from: <https://www.gov.ie/en/publication/6996b-slaintecare-implementation-strategy-and-action-plan-2021-2023/>.

13. Mental Health and Wellbeing Programme (HSE Health and Wellbeing). HSE Social Prescribing Framework Mainstreaming social prescribing in partnership with community & voluntary organisations. 2021 [cited 2024 April 2]. Available from: <https://www.hse.ie/eng/about/who/healthwellbeing/our-priority-programmes/mental-health-and-wellbeing/social-prescribing/>.
