# Supplementary material for: Understanding how intermediaries connect adults to community-based physical activity: A qualitative study
Source: PLoS One. 2025 Jan 31;20(1):e0318687. doi: 10.1371/journal.pone.0318687 (PMC11785267; doi:10.1371/journal.pone.0318687)
Supplement: S2 File — A sample audit trail for the cluster ‘mental health issues negatively impact engagement’, part of the theme ‘connecting individuals to physical activity’. (DOCX) [file pone.0318687.s002.docx]

**S2 File. Sample audit trail**

21/06/2023 13:54

**Clusters and codes**


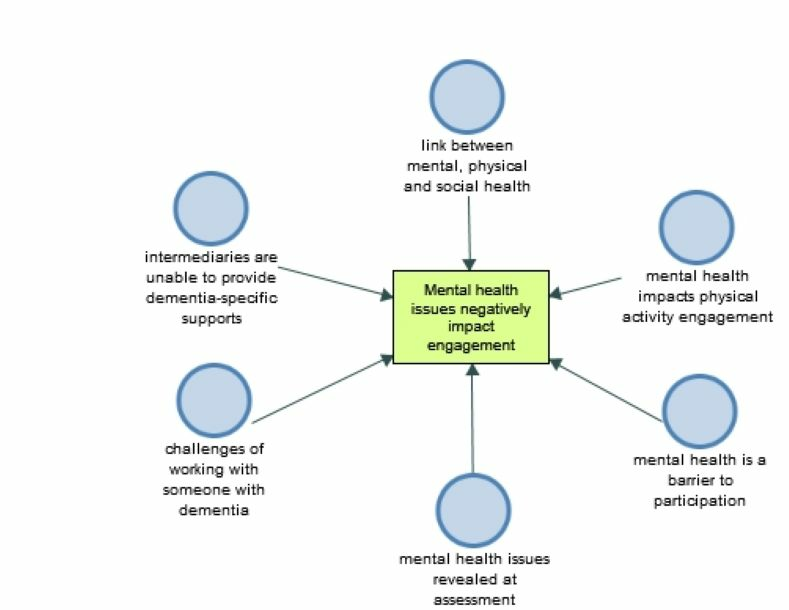


**Visualisations**


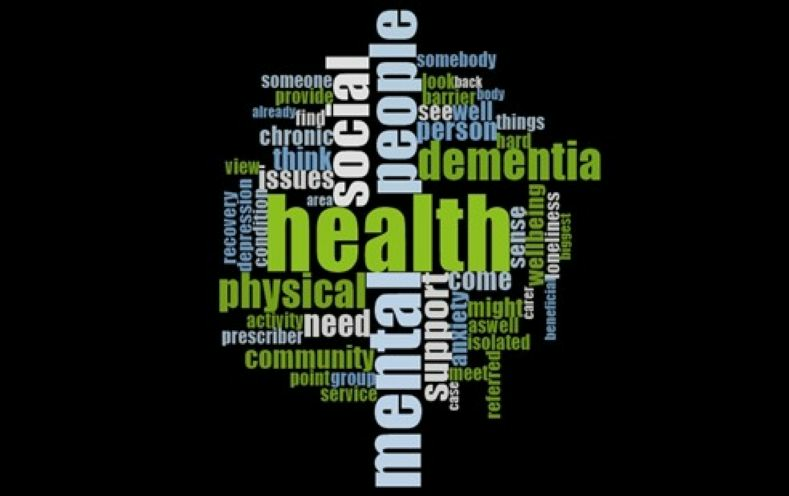


**Links to literature**

"Staff acknowledged how physical activity could help with some conditions (e.g. strengthening the body or some mental health conditions). But it was perceived as a barrier for some, resulting in lower attendance and an inability to participate in certain activities." (i)

"Many individuals with mental ill-health have unmet social needs leading to loneliness [45], with loneliness exacerbating conditions [46]." (ii)

"For instance, one participant, who was discharged due to lost engagement, talks at length about the anxiety she experienced around meeting her link worker. For her, even engaging with the intervention was ‘unthinkable’." (iii)

"Carol was very anxious about going to the gym: “when I went the gym, I sobbed my heart out, cried my eyes out”. Subsequently she was discharged from the intervention due to ‘lost engagement’. At her final interview Carol got extremely upset, recalling how she: “pushed everybody away, i.e. Amy, that was trying to help me and get me on the right track and everything. And I just couldn’t do it.”" (iv)

**Longer cluster description**

Clients of SP (and HPO to some extent) were reported to have more complex needs which were often reported during this assessment which may not have been known to the intermediary in advance, like mental health issues or other psychosocial stressors (v, vi, vii). Both physical and mental health issues were identified by all intermediaries as substantial barriers to both engaging with their service (viii, ix), and engaging in physical activity (x). This could be in relation to starting the process of behaviour change, or trying to be physically active in the context of managing their condition which may fluctuate (xi, xii, xiii, xiv). Despite the challenges posed by these health issues, all intermediaries recognised the value of physical activity and its ability to improve many aspects of health for people living with a chronic disease; physical, mental, and social (xv, xvi, xvii).

**See Also Links**

i Files\\Literature\\A community-based, sport-led programme to increase physical activity in an area of deprivation~ a qualitative case study [1]

ii Files\\Literature\\Therapeutic Community Gardening as a Green Social Prescription for Mental Ill-Health~ Impact, Barriers, and Facilitators from the Perspective of Multiple Stakeholders [2]

iii Files\\Literature\\Social prescribing and classed inequality~ A journey of upward health mobility~ [3]

iv Files\\Literature\\Implementation and impact of a social prescribing intervention~ an ethnographic exploration [4]

v Files\\Interviews\\SOCIAL PRESCRIBING LINK WORKERS\\P07

“It’s more that from that so say on the mental wellbeing tool they answered that they were never relaxed, didn’t feel close to people, none of the time for everything. Em I would know then that there’s possibly if they haven’t said it already maybe depression or anxiety issues or something”

vi Files\\Interviews\\SOCIAL PRESCRIBING LINK WORKERS\\P19

“they might be isolated or have mental health issues but that will frequently come up then aswell.”

vii Files\\Interviews\\SOCIAL PRESCRIBING LINK WORKERS\\P27

“So, a typical case would be Mary goes to see her doctor. Mary says I am struggling, I haven’t left the house since Covid, the doctor writes and says P27 can you see Mary, Mary likes knitting, art, painting and so forth. Mary comes in to the social prescriber and it’s nothing to do with that. It’s to do with loneliness, it’s to do with mental health, depression, low mood and we look at the biopsychosocial model of a person so we look at all of the being and the wellness.”

viii Files\\Interviews\\SOCIAL PRESCRIBING LINK WORKERS\\P03

“some people have quite significant mobility issues em you know where they could have severe social anxiety and things like that so in those cases where people find it really hard to come out and meet me I will go to their home and meet them.”

ix Files\\Interviews\\SOCIAL PRESCRIBING LINK WORKERS\\P21

“So it-it is social prescribing, it is, you know, it's at a certain level. You know it's not going to cure or help with-if somebody’s in a very deep depression. Yes, low mood and anxiety, definitely.”

x Files\\Interviews\\HEALTH PROMOTION AND IMPROVEMENT OFFICERS\\P26

“I don't think the gaps are in policy or in evidence, or in like [laughing] any of that kind of stuff, we know physical activity works, we *know* we all need to do it, and we all know *why*. But I think the biggest barrier is just that-that handholding piece, and someone making sure that what's on offer in any locality or for any specific community group, is tailored to their needs and is accessible, you know, to them, whether the barriers are psychological, physical, or health related.”

xi Files\\Interviews\\LOCAL SPORTS PARTNERSHIP OFFICERS\\P01

“we've already had a request but we're not in a position for that. If somebody with dementia, for example, once, um, how would I say wants something that's eh, very individual. We wouldn't be able to do that to the best of our abilities.”

xii Files\\Interviews\\LOCAL SPORTS PARTNERSHIP OFFICERS\\P29

“They're very hard to reach people, inactive people, very hard to reach, because they-they're inactive for a reason. They're either nervous about coming out, or they don't have-think there's anything out there for them as well.”

xiii Files\\Interviews\\SOCIAL PRESCRIBING LINK WORKERS\\P16

“it’s a very challenging area in that there is such a variety um, such a broad range of dementia, and some are so slow progressing and some can develop so quickly”

xiv Files\\Interviews\\SOCIAL PRESCRIBING LINK WORKERS\\P27

**“Interviewer: That’s ok. And ok so then maybe I’ll rephrase that. Would you think that everyone that is referred to you is kind of suitable to join an exercise group of some description?**

Participant: No. Why would I say that? Because I think it would range from their mental health, their mental wellbeing is a barrier or a challenge for them to engage.”

xv Files\\Interviews\\HEALTH PROMOTION AND IMPROVEMENT OFFICERS\\P15

“Um, always you know, the three-pronged um, approach for me would be, you know, physical health, mental health, and social connectedness, so any initiative that can um, provide those three things”

xvi Files\\Interviews\\SOCIAL PRESCRIBING LINK WORKERS\\P03

“A lot of people with you know trying to manage a physical chronic health condition and an awful lot of people like so everyone who has presented social prescribing in [county] so far through conversation has talked about trying to manage a chronic health condition be it physical, sensory or you know mental health and I just thought that was unbelievable how much their health was impacted and maybe their social engagement as well you know.”

xvii Files\\Interviews\\SOCIAL PRESCRIBING LINK WORKERS\\P18

“typically people who I see are vulnerable. They are isolated in the community. They mightn’t be leaving their house very often. They don't have many connections in the community. They don't have - maybe they don't have particularly strong relationships with friends or family, and also they might have some sort of complex kind of social backgrounds, and typically some form of chronic condition or a mental health kind of issue, and which could obviously be linked or just independent of one another.”

**References**

1. Garner-Purkis A, Alageel S, Burgess C, Gulliford M. A community-based, sport-led programme to increase physical activity in an area of deprivation: a qualitative case study. BMC Public Health. 2020;20(1). doi: 10.1186/s12889-020-08661-1.

2. Wood CJ, Polley M, Barton JL, Wicks CL. Therapeutic Community Gardening as a Green Social Prescription for Mental Ill-Health: Impact, Barriers, and Facilitators from the Perspective of Multiple Stakeholders. International Journal of Environmental Research and Public Health. 2022;19(20). doi: 10.3390/ijerph192013612.

3. Gibson K, Pollard TM, Moffatt S. Social prescribing and classed inequality: A journey of upward health mobility? Social Science & Medicine. 2021;280:114037. doi: 10.1016/j.socscimed.2021.114037.

4. Pollard T, Gibson K, Griffith B, Jeffries J, Moffatt S. Implementation and impact of a social prescribing intervention: an ethnographic exploration. British Journal of General Practice. 2023;73(735):e789-e97. doi: 10.3399/BJGP.2022.0638.
